# Learning physics online or face-to-face: A case study of STEM and non-STEM students

**DOI:** 10.3389/fpsyg.2022.1041187

**Published:** 2022-11-18

**Authors:** Gaydaa Al-Zohbi, Maura A. E. Pilotti, Hanadi Abdelsalam, Omar Elmoussa

**Affiliations:** ^1^College of Sciences and Human Studies, Prince Mohammed Bin Fahd University, Khobar, Saudi Arabia; ^2^Department of Student Affairs, Prince Mohammed Bin Fahd University, Khobar, Saudi Arabia

**Keywords:** STEM, non-STEM, instructional mode, academic success, Middle East

## Abstract

The academic performance of young women is particularly relevant to the success of societies that have only recently begun to address gender inequalities in education and the workforce. The present research examined the performance in a physics course of STEM and non-STEM female freshmen from such a society. It aimed to determine whether the change to online instruction, forced by the pandemic on students who had been accustomed to the face-to-face mode, affected their performance. In the study, performance on lab assignments and tests distributed across the semester (formative assessment measures) differed. Namely, STEM students performed better than non-STEM students on lab assignments and better online than face-to-face on tests. Non-STEM students’ performance on both lab assignments and tests remained insensitive to the mode of instruction. Performance on the final test and course grades, both of which were treated as summative assessment measures, replicated the pattern of effects exhibited by tests distributed across the entire semester. For all students, prior math proficiency made a limited contribution to performance. The findings of this study suggest that young women, who during the pandemic were brought back to the constraints of the home, were resilient in the face of change. According to physics instructors and students, by distributing study efforts more continuously in the online mode and taking advantage of recorded class meetings, they managed to promote performance (as per STEM students) or preserve it (as per non-STEM students).

## Introduction

The pandemic may be an afterthought for many, but a retrospective examination of its impact on performance can offer useful information on how online and face-to-face learning differ, especially for students unaccustomed to the former. Furthermore, in the post-pandemic world, the remnants of the pandemic have not vanished. Most institutions now rely more heavily on the online mode, which they had previously largely shunned for their day-to-day operations (e.g., offering online or blended course options, conducting online meetings, relying on virtual laboratories, etc.), thereby making understanding the impact of mode of instruction on students’ learning even more important.

To assess adaptation to a learning environment that had suddenly changed, a considerable number of studies have focused on self-reports by instructors and students (e.g., Wijaya et al., 2020; Al-Taweel et al., 2021; Biwer et al., 2021; Naujoks et al., 2021). The main assumption underlying these studies has been that academic performance depends on students’ self-regulatory activities (Duncan and McKeachie, 2005; Panadero, 2017; Broadbent and Fuller-Tyszkiewicz, 2018; Biwer et al., 2021), which may broadly include planning, monitoring, and controlling the acquisition of information and skills, as well as applying resource-management strategies (e.g., management of time, attention, effort, and motivation). The ancillary assumption has been that online learning, especially when it is a sudden and forced option, demands more self-regulation than face-to-face learning (Duzgun and Basaran, 2021; Rivers et al., 2022). As such, it may come with the adoption of a more continuous engagement in learning activities (Gonzalez et al., 2020). The evidence brought forth by such studies has suggested that students faced both emotional and cognitive challenges, but also displayed resilience, thereby illustrating different rather than uniform responses to instructional changes (Broadbent and Fuller-Tyszkiewicz, 2018; Biwer et al., 2021). Other studies, also interested in assessing adaptation to a learning environment that had suddenly changed, have examined academic adjustment from the viewpoint of performance, thereby asking if the online mode of instruction had jeopardized learning. The evidence from this line of work has been mixed. Namely, field studies have reported performance improvements (Elzainy et al., 2020; Gallego-Gómez et al., 2020; El Said, 2021; Engelhardt et al., 2021; Iglesias-Pradas et al., 2021; Zheng et al., 2021), declines, or no change at all (AbdelSalam et al., 2021; Foo et al., 2021; Hussain et al., 2021; Zheng et al., 2021). Interestingly, research that has relied on self-reports has tended to highlight individual differences in students’ responses to online learning (Biwer et al., 2021; Pilotti et al., 2022b). In contrast, research that has relied on performance measurements has mostly focused on explaining the observed learning outcomes (including declines, improvements, or stability) by relying upon organizational and technical factors related to the delivery of online courses and their content (Gonzalez et al., 2020; Iglesias-Pradas et al., 2021).

Extant research has also included different educational levels. Concerning high school (a precursor of college-related performance), evidence has been mixed with studies reporting no differences in performance before and after the pandemic, declines, or gains spread across a diverse array of countries. For instance, Tomasik et al. (2021) reported no differences in learning (as measured by formative assessment tools), whereas Kuhfeld et al. (2020) claimed declines in overall performance. Breaux et al. (2022) reported overall performance declines in male but not female students, which they attributed to the higher self-discipline of female adolescents. In a systematic review of the performance of children and adolescents, Panagouli et al. (2021) noticed that although students could experience either declines or benefits, the younger their age, the more likely deficits were to be observed. The examination of particular science-related disciplines has not brought consistency in research findings. For instance, Spitzer and Musslick (2021) and Zhang et al. (2021) found gains in math performance. Turner et al. (2020) found declines in chemistry, and Burkholder and Wieman (2022) as well as Carleschi et al. (2022) reported no difference in physics. Measured against standards of learning outcomes in physics, Melinda et al. (2021) found shortcomings. Yet, Liao et al. (2022) noted that the negative or positive impact of the pandemic on high-school students’ learning was mostly related to parents’ educational level. As the burden of ensuring learning shifted more heavily to parents, the latter’s educational level, through engagement in their children’s academic activities, became quite relevant. Interestingly, compared to the volume of research involving educational levels before college, only a limited number of studies are available that have compared online and face-to-face instructional approaches for university-level introductory physics. Unsurprisingly, their results are mixed. For instance, Fouad et al. (2021) who surveyed students’ performance in a physics course as a function of synchronous (i.e., real-time) online and face-to-face instruction, reported higher performance online. In contrast, Faulconer et al. (2018) reported no significant differences between synchronous online and face-to-face physics in performance, as well as withdrawal rates, and failure rates. Although studies on the impact of the pandemic on learning and teaching have spanned all continents, not much attention has been devoted to college student populations who have lacked prior experience with online learning.

## The present study

The present study specifically focuses on students who had received only face-to-face instruction since the start of their educational journey in elementary school, under the assumption that such students would be particularly sensitive to disruptions of their habitual learning environment. Within this population, the study targets female students of a society in transition from a strictly patriarchal order, which did not grant women autonomy and agency, to one that aspires to meet gender-equity standards. In this kind of society, of which Saudi Arabia (SA) is a prototypical example, women had been relegated to the home, with fewer rights and less autonomy of movement and decision-making than men, for as long as anyone can remember. Only recently, through legislative and financial actions from the top, women have acquired a status equal to that of men in educational practices and professional opportunities (Saqib, 2016). These actions have in turn demanded that women, as much as men, contribute to the economic engine of Saudi society (Esmail, 2018). Of course, drastic changes from the top take time to puncture a social fabric that has been defined by women and men having different rights, obligations, and standards of conduct, and being relegated to different physical spaces inside and outside the home (Meijer, 2010). As a result, the remnants of old social practices, illustrating pride in one’s traditions and customs, coexist undisturbed with the newly acquired social practices (Pilotti et al., 2021a). For instance, since gender segregation in public spaces has been removed as a legal requirement, women and men can work together and mingle freely in public spaces. Women can now pursue educational careers and professions in STEM (Science, Technology, Engineering, and Mathematics) fields, once inaccessible to them (Barry, 2021). As such, they are entering STEM fields in greater numbers than men (Islam, 2017; Narasimhan, 2021), and often show higher academic performance than men (El-Moussa et al., 2021). However, some are still reluctant to enter such fields to avoid challenging traditional gender norms (Islam, 2017; Varshney, 2019). It has been argued that systemic changes from the top require time to be processed by their recipients before they become a widespread reality on the ground (Al-Bakr et al., 2017; Pilotti et al., 2021b). Thus, time may be needed for most women to appreciate that choosing one of such fields is not only possible but also an advantageous course of action (e.g., higher demands and compensation packages).

The impact of the COVID-19 pandemic in SA has unique features if one considers the status of women. The pandemic appeared while top-down social changes were beginning to percolate through the entire social fabric of a society known for its uncertainty avoidance (Cassell and Blake, 2012). It restricted mobility, as well as minimized and regulated social interactions. Thus, for women, the experience of the pandemic might have been exemplified by a return to the confinements of the home, once again deprived of freedom of movement and agency. Although the Ministry of Education quickly moved instruction, traditionally offered face-to-face, to the online mode and provided financial, technical, and pedagogical assistance for a smooth transition, the impact of online instruction on Saudi female students’ learning is of particular interest due to the recency of their newly acquired agency.

Our study focuses on female freshmen who have enrolled in physics courses of the general education curriculum. As for all courses offered by the selected academic institution, physics was delivered face-to-face before the pandemic and online during the pandemic. By and large, students did not have prior experience with online courses but differed in whether their major was STEM or non-STEM. The selection of a major entailed a differential exposure to the sciences in the last 2 years of high school. Namely, STEM majors would include students who had received additional instruction in physics, chemistry, math, and computer science, whereas non-STEM majors would include students who had received additional instruction in the humanities and social sciences (Wiseman et al., 2008; Aloraini, 2020). To acknowledge the differential prior educational experiences of such students and the differential needs of their majors, at the selected institution, introductory physics is separated by major into two parallel courses, one open to STEM students and one open to Non-STEM students. Both courses focus on understanding Newtonian physics, and its practical applications (including data collection, analysis, organization, and interpretation). The fundamental difference between the two courses is that the course for STEM students is calculus-based whereas the course for non-STEM students is algebra-based.

The present study asks the following questions, each presented with its rationale:

- Did STEM students and non-STEM students perform differently in the online and face-to-face modes? It is reasonable to predict that the greater background knowledge in the sciences of STEM students might have better equipped them to deal with changes in their learning environment, thereby promoting adaptation and thus performance. Alternatively, the greater background knowledge in the sciences of STEM students might have fostered overconfidence in the face of changes in the learning environment, thereby preventing adaptation and impairing performance. Evidence exists that overconfidence includes a failure of metacognition (i.e., reflecting on and critically examining one’s own thinking and actions; Hall and Sverdlik, 2016). As such, overconfidence can impair students’ self-regulation of learning activities due to overestimation of how much they have learned. Consequently, they are likely to neglect self-monitoring, reflection, and realistic goal-setting, all of which are critical to academic success (Stone, 2000). Interestingly, overconfident students have been reported to be significantly more prevalent in STEM disciplines than in non-STEM disciplines (Reuben et al., 2017).
- Did math proficiency matter differently in online and face-to-face courses? In physics courses, an important contributor to performance is assumed to be math proficiency (Hudson and Rottmann, 1981; Konvalina et al., 1983; Hart and Cottle, 1993; Bautista, 2013; Redish, 2021), thereby potentially serving as a relevant buffer for disruptions in the learning format to which both STEM and non-STEM students had been accustomed since an early age. Thus, we first measured the contribution of math proficiency (as measured by prerequisites) to performance in physics and then asked whether the impact of the mode of instruction (face-to-face and online) on students’ performance would change if the contribution of math proficiency were to be statistically removed (*via* analysis of covariance).
- Were particular assessment measures differentially sensitive to instructional changes? Physics courses are usually devoted to conceptual knowledge and practical problem-solving activities where conceptual knowledge is exercised through applications. At the selected institution, physics is taught using an inquiry approach. The assessment of students’ course performance involves, by and large, tests and lab assignments (i.e., reports) distributed across the semester, serving as formative assessment measures, and a final examination, serving as a summative assessment measure. Formative assessment tools are used to measure knowledge and skills limited to specific areas covered by a course, as well as to offer periodical feedback to inform students’ self-regulatory activities (Lau, 2016). They are considered critical to learning as they impact students’ self-perceptions regarding the gap between desired and current knowledge as well as the actions taken by students to close the gap (Tay, 2015). As such, they offer a longitudinal, more fine-grained perspective of learning. Summative assessment, instead, is intended to measure students’ learning comprehensively to determine the quality of knowledge and skills acquired across the entire semester (Taras, 2009). As such, summative assessment offers a one-time window into students’ learning. Some evidence exists that formative and summative assessment measures may be differentially sensitive to the mode of instruction (face-to-face versus online; AbdelSalam et al., 2021), but evidence from physics courses is absent.

At the selected institution, all forms of assessment tend to involve higher-order thinking skills, which refer to an array of skills that loosely correspond to the levels of Bloom’s taxonomy that are above mere comprehension (Bloom, 1976). Instances of higher-order thinking skills in physics may consist of the formulation of research questions and related hypotheses, data analysis, and interpretation of data to draw conclusions (Zohar et al., 1998). Yet, lab assignments are reports regarding specific demonstrations or experiments that students have carried out or have witnessed, which require active data collection, analysis, organization, and interpretation. Tests require an understanding of physics and its implications from described experiments and practical scenarios. Furthermore, although assignments have a deadline, they are allocated more time for completion than tests and are usually carried out at home.

It is reasonable to assume that although both lab assignments and tests demand an understanding of key concepts and the exercise of a problem-solving mindset, lab assignments might not be as sensitive to changes in the mode of instruction as tests since they are carried out at home in both online and face-to-face courses (Kennelly et al., 2011). However, tests and the final examination might display more readily the impact of changes because they measure performance within a restricted time frame, and thus they are more likely to induce anxiety in the test taker. Evidence exists (Yerkes and Dodson, 1908; Cofer and Appley, 1967; Wiener et al., 1984; Dickman, 2002; Corbett, 2015; Piefke and Glienke, 2017) that physiological arousal is beneficial to performance when test demands are low (i.e., easy tests), but it is detrimental to performance when test demands are high (i.e., challenging tests). Thus, if students’ acquisition of physics has been impacted by disruptions of their habitual learning environment, test performance rather than lab performance might be likely to display declines.

## Materials and methods

### Participants

The participants were 813 female students enrolled in either a physics course for STEM majors (PHYS 1421) or non-STEM majors (PHY 1411). Courses were offered either online (during the pandemic) or face-to-face (before the pandemic) to freshmen and sophomores by a Saudi university conforming to a US curriculum and student-centered pedagogy. Students’ prior formal educational experiences did not involve online learning. Both PHYS 1421 and PHYS 1411 are four-credit courses, meeting 3 h per week for lectures, demonstrations, and class discussions, and an additional hour of lab activities. Three semesters were selected for each mode of instruction. Among STEM majors, 263 completed the course online and 277 face-to-face, whereas, among non-STEM majors, 152 completed the course online and 121 face-to-face.

At the selected university, non-STEM majors included students majoring in business, law, and interior design, whereas STEM majors included students majoring in engineering, computer science, and architecture. Instruction, which relied on textbooks imported from the United States, was delivered in English, albeit students were Arabic-English bilingual speakers. Courses were taught face-to-face before the pandemic and online during the pandemic by one of three instructors, all of whom had at least 5 years of prior higher education instruction and advanced degrees in the field. Instructors were a constant across courses and modes of instruction, thereby minimizing the impact of instructors’ idiosyncrasies in comparisons involving major and mode of instruction. Most importantly, the quality assurance policies of the selected university required that instructors for each course conform to equivalent standards in the content and delivery of the curriculum, and in the assessment of learning. The curriculum and the assessment protocol of all general education courses, including introductory physics, had been developed and approved by the Texas International Education Consortium (TIEC), which periodically would perform quality assurance visits. Course coordinators whose responsibilities entailed monitoring teaching and assessment of learning ensured compliance with quality assurance policies.

### Materials and procedure

At the selected institution, physics is taught using an inquiry approach that is intended to promote active learning, including critical thinking and problem-solving practices. Both PHYS 1411 and PHYS 1421 rely on the acquisition of theoretical knowledge of physics and require practical problem-solving activities where conceptual knowledge is exercised through applications. Math prerequisites involve an intermediate algebra course for PHYS 1411, and a pre-calculus course for PHYS 1421.

It is important to note that each course was taught exclusively face-to-face before the pandemic and online synchronously during the pandemic. For students, the synchronous virtual classroom was a novel environment, albeit they were familiar with an e-learning platform (e.g., Blackboard), which had been used in face-to-face classes to access and submit course materials. The synchronous virtual space replicated many aspects of the face-to-face space (Dennis, 2003). The virtual classroom and lab were equipped with Blackboard Collaborate, which is a video conferencing tool that allowed students to have real-time interactions as if they were enrolled in a face-to-face course. Online, instructors were required to meet the standards set by Moore (1989) as necessary for successful online education. That is, in the classroom and outside the classroom (i.e., virtual office hours), they were to maximize learner-content interaction, learner-instructor interaction, and learner-learner interaction. As per the face-to-face mode, Blackboard gave students access to study materials and resources, such as study guides, textbooks, and videos. Each lecture was recorded so that students could access it later if they needed reiteration. During online tests, students’ activities were supervised by instructors through cameras. A lock-down browser application (Respondus) was required for tests. All assignments were scrutinized with anti-plagiarism software.

Each physics course was organized into two main categories of formative assessment, each consisting of four lab reports and four tests. A summative assessment, which entailed a final examination at the end of the semester, terminated each course. Class performance was computed as a weighted mean, including lab assignments (25%), tests (50%), and the final examination (25%). Physics grades for labs, tests, final tests, and course grades were obtained from instructors. Prior Math scores were obtained from the Office of the Registrar as letter grades. Because the selected physics courses required the fulfillment of a math prerequisite, all scores involved grades equal to D+ (i.e., the minimum score to pass a course at the selected institution) or greater. Letter grades were translated into numerical values for use in statistical analyses. The middle point of the range of values that each letter grade represented was used as the math grade that a given student received: D+ = 67.5%, C = 72.5%, C+ = 77.5%, B = 82.5%, B+ = 87.5%, A = 97.5%, and A+ = 98.0%. Overall class performance, as measured by class grades, was treated as an indicator of summative assessment, which illustrated students’ performance across a broader timeframe than the 2 h allocated to the final test.

Important to note that for a subset of STEM students (*n* = 141) and non-STEM students (*n* = 129) of the current study, data about self-efficacy beliefs (confidence in one’s abilities; Chen et al., 2001) were available from internal assessment protocols instituted by instructors of the general education curriculum to identify suitable interventions for at-risk students. Such students had completed a general self-efficacy questionnaire (Chen et al., 2001) either before the pandemic or during the pandemic. However, records only allowed for the determination of whether they served as participants in our study without links to their performance. Based on the evidence collected from a sub-sample of participants, no difference between STEM (*M* = 3.75; *SD* = 0.97) and non-STEM students (*M* = 3.66; *SD* = 1.00) was found [range 1–5; *F*(1, 268) < 1, *ns*]. The same null findings were obtained in another study involving math learning (Pilotti et al., 2022a). Thus, the variable self-efficacy was not included in the quantitative analyses listed below.

## Results

Descriptive statistics of students’ performance by major (STEM and non-STEM), mode of instruction (face-to-face and online), and type of assessment are displayed in Table 1. In the table, prior math performance is reported in the last row.

**Table 1:** Descriptive statistics (means and standard deviations) as a function of major and mode of instruction.

	Non-STEM FtF	Online	STEM FtF	Online
*Formative assessment*				
Lab reports	77.27% (13.19)	78.78% (12.75)	88.26% (12.30)	87.81% (11.95)
Tests	78.64% (10.38)	76.74% (14.99)	75.75% (14.64)	80.23% (12.00)
*Summative assessment*				
Final test	67.10% (15.36)	69.84% (16.08)	67.37% (20.99)	76.58% (19.92)
Course grade	75.41% (9.78)	75.52% (11.56)	76.79% (12.11)	81.21% (10.88)
*Prior math performance*	76.10% (8.97)	78.20% (11.06)	75.68% (11.56)	78.40% (13.03)

FtF – face-to-face.

### Were there performance differences?

The analyses described below were intended to answer the following research questions: Did STEM students and non-STEM students perform differently in the online and face-to-face modes? Were particular assessment measures for such students differentially sensitive to instructional changes? The skewness of the data (Cohen, 2008) was below 2 (Curran et al., 1996).

A 2 (mode of instruction) × 2 (major) between-subjects analysis of variance (ANOVA) was conducted on each assessment measure to determine whether there were group differences. The findings of inferential statistics were considered significant at the 0.05 level. Table 2 illustrates that formative assessment measures behaved differently. Lab assignments only showed the main effect of the major selected by students. Namely, STEM students exhibited higher performance both online and face-to-face than non-STEM students but no sensitivity to the variable instructional mode. Tests, instead, displayed a significant interaction between the mode of instruction and major. Tests of simple effects indicated that the test performance of non-STEM students was not sensitive to the mode of instruction [*t* < 1.19, *ns*], whereas the test performance of STEM students was higher online [*t* (538) = 3.88, *p* < 0.001].

**Table 2:** The results of the 2 (Mode of Instruction) × 2 (Major) A NOVA on formative and summative assessment measures.

	*F*	*Df*	*MSE*	*p*	Partial Eta^2^
*Main effect of mode*					
Lab reports	<1	1, 809	153.95	*ns*	
Tests	1.67	1, 809	177.69	*ns*	
Final test	17.73	1, 809	362.02	<0.001	0.021
Course grade	7.26	1, 809	127.48	0.007	0.009
*Main effect of major*					
Lab reports	117.15	1, 809	153.95	<0.001	0.126
Tests	<1	1, 809	177.69	*ns*	
Final test	6.11	1, 809	362.02	0.014	0.008
Course grade	17.59	1, 809	127.48	<0.001	0.021
*Interaction*					
Lab reports	1.12	1, 809	153.95	*ns*	
Tests	10.305	1, 809	177.69	0.001	0.013
Final test	5.20	1, 809	362.02	0.023	0.006
Course grade	6.58	1, 809	127.48	0.011	0.008

MSE = Mean Squared Error; *p* = significance level; Partial Eta^2^ = The proportion of variance uniquely accounted for by each factor or interaction.

Summative assessment measures behaved similarly. Specifically, for the final test, there was a main effect of mode, a main effect of major, and a significant interaction. As per tests distributed across the entire semester, non-STEM students exhibited no performance differences as a function of mode [*t* < 1.43, *ns*], whereas STEM students exhibited higher performance online [*t* (538) = 5.23, *p* < 0.001].

Course grades displayed a main effect of mode and a significant interaction. As per tests distributed across the entire semester and the final examination, non-STEM students exhibited no performance differences as a function of mode [*t* < 1.00, *ns*], whereas STEM students exhibited higher performance online [*t* (538) = 4.46, *p* < 0.001].

### Did math proficiency matter?

To determine the extent to which prior math scores might modulate physics performance, we first examined the extent to which math proficiency predicted performance in physics. Table 3 illustrates the significant Pearson correlation coefficient between assessment measures and math proficiency as a function of academic major and mode of instruction. The correlations are not very different in magnitude from those reported by Hudson and Rottmann (1981) who focused on students’ performance in algebra-based physics and relied on a diagnostic test of mathematical skills to determine math proficiency. In the present study, coefficients of determination ranged from 2.13 to 14.67%, thereby indicating that the contribution of math proficiency to students’ performance in physics was rather modest.

**Table 3:** Pearson correlation coefficients for each major and mode of instruction.

	Non-STEM FtF	Online	STEM FtF	Online
*Formative assessment*				
Lab reports	+ 0.305	+ 0.248	+ 0.185	+ 0.146
Tests	+ 0.249	+ 0.227	+ 0.394	+ 0.245
*Summative assessment*				
Final test	+ 0.284	+ 0.327	+ 0.226	+ 0.326
Course grade	+ 0.347	+ 0.330	+ 0.383	+ 0.324

FtF – face-to-face.

To assess the impact of academic major and instructional mode without prior math scores as a contributor, we then conducted the *F* tests described above with math proficiency as the covariate (i.e., analysis of covariance, otherwise known as ANCOVA). The pattern of outcomes yielded by ANOVA did not change when math proficiency was statistically removed *via* ANCOVA. The exception was course performance, which no longer displayed the main effect of academic major [*F* = 3.12, *ns*].

## Discussion

The findings of the present research were shared with instructors and students to ensure the inclusion of their viewpoints. Comments were anonymized (except for major: STEM versus non-STEM) and organized thematically by two independent raters. The most frequent comments were then used to inform the interpretation of the quantitative results. According to Silbereisen and Tomasik (2011), events that are located in the macro context of a student’s ecosystem become relevant and impactful when they translate into his/her proximal micro-ecosystems, such as the ordinary academic life of the student before the pandemic. Within these micro-ecosystems, the student’s habits and routines are disturbed by such events and require some adaptation. The present study relies on this conceptual framework for assessing performance differences before and during the pandemic (*via* quantitative analyses of students’ grades) and understanding their sources (*via* qualitative analyses of comments made by instructors and students).

Findings could be summarized in three main points. First, as expected, lab assignments, which were carried out mostly at home in both online and face-to-face classes, were not sensitive to the mode of instruction. STEM students had higher grades than non-STEM students in both instructional modes, a difference that instructors attributed to the broader coverage of physics that STEM students received in the last 2 years of high school. Indeed, although the first of the last 3 years of high school included instruction in the natural sciences for all students, STEM students devoted the remaining 2 years to the natural sciences, including physics, whereas non-STEM students allocated the same number of years to the humanities and social sciences. The insensitivity of lab performance to instructional mode did not surprise instructors who judged it as illustrating that their efforts to equate the online and face-to-face classes in content and pedagogy had been fruitful. It did not surprise students either since lab reports of in-class experiments and demonstrations were carried out largely at home in either online or face-to-face classes.

Second, test performance was sensitive to the mode of instruction, but not for all students and in a manner that both instructors and researchers did not expect. Namely, only STEM students displayed the effects of mode of instruction on test performance. Furthermore, contrary to the prediction that the online mode would exhibit the effects of disruptions of learning relative to the habitual face-to-face mode, STEM majors’ online performance was found to be higher than face-to-face performance. On the other hand, non-STEM students were largely insensitive to changes in the mode of instruction, even though they were expected to be more sensitive to such changes due to their lower background knowledge in physics arising from their choice of the humanity and social science track in high school.

At first blush, the findings of our research can be said to be consistent with those illustrating that in the presence of substantial institutional and instructional support students may not exhibit evidence of learning declines in the online mode (as per non-STEM students) or may even display gains (as per STEM students; Elzainy et al., 2020; El Said, 2021; Engelhardt et al., 2021; Iglesias-Pradas et al., 2021). They add to the extant literature that has examined the impact of the Covid-19 pandemic on academic success a focus on academic majors, and the inclusion of an understudied population of students who had not been accustomed to online learning. In this respect, our findings offer a window into young women of a society in transition who displayed resilience in the face of the restrictions that the pandemic imposed on them. The women in our sample were not crashed by the pandemic, even though it reminded them of a return to a time of less mobility and agency. Their comments during debriefings contained reports of a more continuous distribution of study activities, often attributed to the extra time that was available to them since they did not have to make laborious travel arrangements, travel to the university, and dress up to meet others (see also Gonzalez et al., 2020; Iglesias-Pradas et al., 2021). In addition, students mentioned that the online mode allowed them to record class meetings and then listen to the recordings for reiteration and clarification of matters. More continuous studying when classes were offered online along with the opportunity for reiteration and clarification arising from recordings of class meetings might have been responsible for preserving performance levels in non-STEM students, whereas it might have improved performance in STEM students as it grew out of a broader knowledge background and interest in the sciences.

Third, math proficiency predicted performance in physics, but its contribution across academic majors and modes of instruction was rather modest. In agreement with Hudson and Rottmann (1981), our findings suggest that besides math competency, other factors are likely to be more influential, such as the general confidence that students exhibit in their abilities to overcome obstacles and complete tasks given to them (i.e., general self-efficacy; Chen et al., 2001; Bouih et al., 2021). In another study involving math learning (Pilotti et al., 2022a), we found no differences in female students’ general self-efficacy between STEM and non-STEM majors. The values that students attributed to themselves as indices of their self-efficacy beliefs were substantial across the board though. The same null outcome was obtained from a subset of students of the current study for whom self-efficacy beliefs were available from internal assessment protocols instituted by instructors of the general education curriculum to identify suitable interventions for at-risk students. Such students had completed a general self-efficacy questionnaire (Chen et al., 2001) either before the pandemic or during the pandemic as part of the aforementioned protocol. Yet, comments made by students and faculty, which were used to inform the interpretation of the quantitative results, implied that self-efficacy beliefs were given a different role depending on the academic major. Namely, for STEM students, it was likely to serve as a motivator to confront challenges that might hurt academic performance (e.g., doing well in an online physics course), thereby accounting for their higher performance in online physics. Instead, for non-STEM students, self-efficacy was likely to serve as a mere buffer against instructional changes that might hurt their performance, thereby accounting for the absence of an impact of mode of instruction on this group of students in physics.

The present research has limitations, which are to be addressed in future research. First, it is noteworthy to mention the lack of a quantitative assessment of students’ test anxiety, albeit anecdotal evidence collected during office hours and debriefing suggests the presence of anxiety related to test performance specifically in the sciences. Second, female college students from SA may be in a privileged position compared to other Middle Eastern students. They receive substantial financial support and are given a considerable range of academic resources to support their educational endeavors (e.g., state-of-the-art facilities, functional online platforms, easy reliance on technical assistance, internet services fitted to their needs, etc.). Thus, the impact of the pandemic on educational activities may be weaker than in other student populations. Third, it may also be weaker due to female students’ engagement, which is driven by their intense desire to succeed professionally. Thus, the impact of engagement on learning needs to be explored further. To this end, it is important to consider three dimensions of academic engagement: academic self-confidence (i.e., self-efficacy), academic self-reliance (i.e., trust in the available resources to accomplish any number of tasks), and connectedness (Coates, 2006), all of which might shape the academic performance of young women in a society attempting to bend patriarchal norms and customs to gender equity standards. Fourth, the role of the acquisition of physics in a second language needs to be considered. The participants of our study belong to a student population for whom Arabic was the primary means of instruction in high school, whereas English became the dominant language in college. At the selected institution, proficiency in the English language is demonstrated at the time of enrollment, and further practice is given in each of the courses offered by the university (either in the general education curriculum or in the curriculum of the selected major). Nevertheless, the challenges that students may face in encountering physics in English for the first time add a burden to the adaptation that freshmen need to make to college life. The challenges of adaptation, including the processing of study materials in a second language, deserve further scrutiny. Fifth, although STEM and non-STEM students differed in the extent of their previous coverage of scientific disciplines in high school, no information was available regarding their actual performance in high-school physics courses. Thus, we were unable to examine the predictive validity of physics pre-college preparation. Evidence supporting the relationship between high-school physics preparation and college performance in physics ranges from a weak or modest relationship (Halloun and Hestenes, 1985; Hart and Cottle, 1993; Alters, 1995; Burkholder and Wieman, 2019; Hewagallage et al., 2022), to no relationship at all (Champagne and Klopfer, 1982). The extant literature offers an array of factors that may modulate this relationship and its strength, including the quality of high-school physics instruction, the extent of physics preparation (e.g., one course or more than one), and even students’ overall high-school achievement (Sadler and Tai, 2001; Bazelais et al., 2018; Lawton et al., 2021). The relationship between high-school physics and performance in introductory college physics is a complex matter that is to be examined in future research.

## Conclusion

The findings of the present study illustrate two main issues that are relevant to the literature on students’ adaptation to crises (i.e., sudden changes in the status quo; Biwer et al., 2021) and the learning of physics. First and foremost, it is noteworthy to point out that while the female students of our study, who were accustomed to face-to-face learning, might have experienced challenges in the online mode due to its heavier demands for self-regulation, their responses exhibited resilience. Faculty and students attributed resilience to the institutional and instructional support they received at the onset of and during the crisis. Because the online mode is likely to remain a component of ongoing and future educational formats, responses to crises, such as the one we documented here, may serve as frameworks for introducing changes to educational practices in other parts of the world to ensure their sustainability.

Second, the present findings have implications for the learning of physics by illustrating that math, although a contributor to performance, is not the main one. Thus, they suggest that even physics taught through an inquiry approach relies on learners’ resources that are not assessed by an examination of mathematical skills alone (e.g., motivation to succeed in fields previously the sole domain of men). Since the methodologies and topics of physics are deeply ingrained in all sciences and engineering, learners who cannot complete physics successfully may find other science courses particularly challenging (Hudson and Rottmann, 1981). As such, not only institutional and instructional support but also the identification of individual differences that predict physics performance may ultimately be key to the success of students in STEM fields.

## Data availability statement

The raw data supporting the conclusions of this article will be made available by the authors, without undue reservation.

## Ethics statement

The studies involving human participants were reviewed and approved by PMU Deanship of Research. Written informed consent for participation was not required for this study in accordance with national legislation and the institutional requirements.

## Author contributions

GA-Z, MP, HA, and OE equally contributed to the design of the study, to the collection, analysis, and interpretation of the data, as well as to the drafting and proofreading of the article. All authors contributed to the article and approved the submitted version.

## Conflict of interest

The authors declare that the research was conducted in the absence of any commercial or financial relationships that could be construed as a potential conflict of interest.

## Publisher’s note

All claims expressed in this article are solely those of the authors and do not necessarily represent those of their affiliated organizations, or those of the publisher, the editors and the reviewers. Any product that may be evaluated in this article, or claim that may be made by its manufacturer, is not guaranteed or endorsed by the publisher.
